# Cours Théorique et Pratique d'Accouchemens

**Published:** 1834-07-01

**Authors:** 


*Cours Théorique et Pratique d'Accouchemens.* Par J. Capuron, Docteur en Médecine de la Faculté de Paris, Professeur de l'Art des Accouchemens et des Maladies des Femmes et des Enfans, &c. *Sixième Edition.*—Bruxelles, 1832. 8vo. pp. 252.

This work is divided into three parts: the first treats of the anatomy and physiology of the female organs of generation, in their unimpregnated and impregnated state; the second, of natural labour; and the third of preternatural labour, including those cases which require only the assistance of the hand, as well as those which demand the use of instruments.

A French *Cours d'Accouchemens* is generally less comprehensive than an English System of Midwifery. Among our continental neighbours the obstetric art is considered merely as a branch of operative surgery, and treated of apart from the diseases of the reproductive organs, and those consequent on pregnancy and parturition: we doubt, however, whether such insulation be favourable to the progress of the science, since many of the most important and difficult questions which present themselves to the accoucheur are essentially connected with the consideration of disease. The evidences of pregnancy, for example, in a healthy woman, are usually sufficiently unequivocal; but this state is liable to be complicated with diseases which render its diagnosis extremely obscure, and there are also diseases which simulate pregnancy so closely, that all the science and assiduity of an experienced practitioner is requisite for their discrimination. In treating of this question, M. Capuron gives an excellent view of the ordinary means of ascertaining whether a woman be with child or not; but he makes only very casual allusion to the several morbid states, which may render the problem of difficult solution. Our author's remarks on this subject will, however, be read with considerable advantage.

In reviewing the diagnostic signs of pregnancy, he expresses himself with more diffidence of their validity than is usual with obstetrical writers; not, however, with more than the experience of practitioners, if candidly stated, will amply warrant.

"None but an experienced and intelligent accoucheur is capable of separating truth from falsehood in an inquiry where self-deception is so easy; he alone can distinguish the probable, equivocal, or uncertain signs from those which are true, characteristic, and infallible, and thus pronounce an irrevocable decision. In general, whenever the movements of the fœtus cannot be detected, we must be content to remain in doubt till time clears up the difficulty. But it may happen that the obscurity is not dispelled till a very late period, not even till that of actual delivery. We could not have believed this, had we not been witness to the following fact:

"A woman was received at the Hôpital de la Charité, declaring herself to be dropsical. Corvisart examined her, and thought that he perceived a small fœtus towards the left flank: this induced him to believe that the patient laboured under an encysted dropsy complicated with extra-uterine pregnancy. Baudelocque, being requested to visit this woman, examined her per vaginam, and assured us that the appearance of pregnancy was caused by an enormous scirrhus of the uterus. What happens? Fifteen days or three weeks after, the woman is naturally delivered of a very large child, full of vigour and health. What a surprise for the clinical professor! what a warning for the celebrated accoucheur! what a lesson for the pupils! Thenceforth we learned never to decide, under similar circumstances, till we had attained complete certainty of the existence of a fœtus in the womb. By such circumspection alone can we guard ourselves against error, and avoid these stupid mistakes, which tend to the disgrace of the art, to the dishonour of the practitioner, to the injury of the woman, and almost always to the destruction of her offspring. But how are the motions of the fœtus to be excited? This is, perhaps, the most embarrassing part of the subject, at least to those who are only commencing the practice of manual examination. The following, however, are the precepts given for the diminution of the difficulty, if indeed any precepts can supply the want of experience in an art which is only to be acquired by practice. The uterus is to be alternately elevated and depressed, by the application of one hand to the surface of the abdomen, while the index-finger of the other is introduced into the vagina. By this procedure a shock is communicated to the fœtus, which is first carried to the fundus of the uterus, and then suffered to fall towards the neck of this viscus, and made to impinge on the finger, which is kept immoveable in its situation: this precaution is necessary to avoid confounding the passive motion of the fœtus with that of the entire uterus. The percussion felt is stronger in proportion as pregnancy is further advanced, since the product of conception is then more fully developed. In the latter months, the fœtus, when raised, returns quickly upon the finger, and affords unequivocal signs of its presence. Sometimes, also, in carrying the hand over the surface of the abdomen, or applying the finger to the segment of the uterus corresponding to the vagina, we are fortunate enough to excite the active or muscular motion of the fœtus, and then no doubt remains of its being alive." (P. 52.)

M. Capuron is not sanguine in his expectations of the aid to be derived from auscultation in the detection of pregnancy.

"Of late," says our author, "physicians have maintained that the stethoscope of Laennec may serve for the detection of pregnancy, when the fact is doubtful. According to them, this instrument, applied to the hypogastrium of the pregnant woman, transmits distinctly to the ear of the inquirer the sound of the circulation both in the placenta and the fœtal heart. But if there be cases in which this species of auscultation is infallible, how many are there also in which it is fallacious and useless! In proof of this, we appeal to the testimony of enlightened accoucheurs who have made repeated experiments on this subject. If they are candid, they will acknowledge that the wooden cylinder never deserves the preference over the touch of an experienced finger. For our own part, observation has convinced us that the stethoscopic diagnosis of pregnancy stands on the same ground with that of thoracic diseases; and we can assure the reader, without fear of being confuted, that its utility has been exaggerated in both instances." (P. 50.)

On this subject we may safely say, that when the pulsations of the fœtal heart can be distinctly heard, they afford a very unequivocal indication of pregnancy, inasmuch as the sound is of a kind little liable to be mistaken; but we should be extremely sorry to declare that a woman was not with child merely because they could not be heard.

With regard to the placental sound we hardly know what to say; but we will not pronounce it inaudible, because we wot of a man who heard a mouse trotting on the top of a high tower when the said deponent was at the bottom; and moreover, the limits of possibility seem to be daily extending, so that we have quite given up disbelieving, as unphilosophical and Judaical withal.

The directions for the conduct of labour, whether natural or otherwise, are copious, and for the most part excellent. In treating of the former, however, we do not think our author has insisted sufficiently on attention to the state of the bladder. Every experienced accoucheur is aware that this organ sometimes becomes dangerously distended during labour, without the patient being herself conscious of it; attendants also are liable to be deceived by mistaking the discharge of the liquor amnii for that of urine; and, in the advanced stage of labour, a vast quantity of urine is often accumulated in the bladder in a very short space of time, offering an impediment to the descent of the child's head, which is certain to increase the pain, and protract the duration of labour, to say nothing of the danger of irreparable mischief to the bladder itself. We have witnessed a case in which the urine had been freely passed till within a few hours of delivery, and in which the patient did not complain of any uneasiness from its retention: this patient fell a victim to peritoneal inflammation, and on dissection it was found that the bladder had given way at its upper part, and allowed the urine to escape into the cavity of the abdomen.

It cannot be too strongly impressed on the mind of the practitioner, that he ought never to trust, in a matter so important, to the account given by the patient, or her attendants, but should insist on seeing the quantity of urine passed, and examine, from time to time, the state of the bladder, through the parietes of the abdomen and vagina, when, if there be the smallest reason to suspect that the bladder is distended, the introduction of the catheter should be urged, notwithstanding the frequent repugnance of the patient to this operation. We may add, that the accoucheur should never go to a case of labour unprovided with a flexible gum catheter of a small calibre; since, when the cavity of the pelvis is fully occupied by the fœtal head, the introduction of the metallic instrument will often be found difficult and painful, and sometimes impracticable.

In his directions for the management of hemorrhage, M. Capuron makes no mention of the secale cornutum as a means of arresting it, or of the transfusion of blood, with a view to repair the waste of the vital fluid. The former omission is, we think, no great loss; but the subject of transfusion is one of considerable importance, and ought not to have been overlooked in a practical treatise on midwifery.

Our author makes but a brief allusion to the use of the secale cornutum in cases of deficient uterine action, and recommends it chiefly on the authority of others.

"Dr. Villeneuve, one of our estimable and modest colleagues, has published an excellent memoir on this substance, in which he has proved historically that it has been administered with success in six hundred out of seven hundred and twenty cases of parturition. But what other therapeutical agent could cite more, or even an equal number of chances in its favour? There is then nothing to prevent its employment, or, at least, a trial of its powers. The memoir just mentioned may be consulted for the preparations, doses, and modes of administration. The conditions necessary for its application will also be found there; they are the same, with reference both to the mother and child, as those which obtain in natural labour. From which it results, that the spurred rye is an excitant which should only be used to restore the languid or suspended pains of labour, when the uterus, from its inactivity, is inadequate to the expulsion of the fœtus." (P. 101.)

Whether the ergot possesses the power over the uterus which many attribute to it, is a question which we must leave to the decision of those who have had larger experience of the matter than we can pretend to: in prosecuting the inquiry, however, the maxim of Lord Bacon should not be lost sight of, "Is humano intellectui error est proprius et perpetuus, ut magis moveatur et excitetur affirmativis quam negativis."

Admitting, however, that the remedy possesses all the virtues which its advocates claim for it, we do not think it can ever be of very extensive use in the practice of midwifery. If labour be protracted merely from insufficient activity of the uterus, we should in most cases be disposed to advocate the cause of patience *versus* the ergot; if, on the other hand, an impediment arise from even the smallest disproportion between the dimensions of the head and those of the pelvis, we cannot conceive anything more dangerous than the administration of this medicine, since it is obvious that, by increasing the expulsive efforts without diminishing the obstacle to be overcome, it is quite as likely to rupture the uterus as to expel the child: and why, in such a case, should we have recourse to a doubtful or dangerous auxiliary, when we possess one which is both safe and certain, in that old-fashioned but very useful instrument, the forceps?

Nevertheless, as M. Capuron observes, where there is mere deficiency of uterine contraction, there is no objection to the use of the ergot; and it is worth while to try it on a large scale, in order to ascertain whether it really possess the alleged property.

The following observations on *puerperal convulsions* will be read with interest.

"When a woman in labour is affected with convulsions, our first care should be to discover their cause, and to obviate it, if possible. If they be connected with a state of plethora, if there be pain and sense of weight in the head, somnolency, vertigo, tinnitus aurium, the appearance of excitement, and sparkling of the eyes, blood-letting is indispensable, to prevent engorgement of the brain, and compression of the origin of the nerves. But what vein ought to be opened in such a case? The abstraction of blood from the foot has been recommended, but it has not been as successful as that from the neck, or the arm. If the convulsions arise from irritation of the primæ viæ, if there be pain above the eyebrows, with nausea and bitter taste in the mouth, if the tongue be foul and red, and the epigastrium painful, recourse must be had to antiphlogistic remedies, such as local bleeding, and water, either pure, or slightly emulsionated.* If the patient be of a highly nervous temperament, subject to hysteria, or any other spasmodic affection, antispasmodics are to be administered, such as baths, orange-flower water in a large dose, the mineral anodyne liquor of

* *L'eau émulsionnée* means distilled water impregnated with the four cool seeds.

Hoffman, camphor, musk, assafœtida, ether, some preparations of opium, &c. We have twice attended a lady who was afflicted during labour with violent convulsions; she was no sooner immersed in tepid water than she was delivered as if by enchantment. The moral emotions which have been known to occasion convulsions demand bodily repose, tranquillity of mind, and abstraction from the causes of mental excitement. The state of debility arising from excessive hemorrhage requires restoratives, and analeptics, strong broths, and even some spoonfuls of old wine. If the neck of the uterus, from being either too rigid or too irritable, offers undue resistance, and causes the commotion of the nervous system, we must endeavour to relax it, and diminish its sensibility, by exposing it to the vapour of warm water, or placing the patient in a hip-bath, or injecting emollient enemata through the vagina. If, notwithstanding all these means, the orifice of the uterus refuses to yield, and presents a hard, tense, and callous margin, as if in a state of scirrhus, its division becomes necessary under pain of compromising the life of the woman. Dubosc, professor in the surgical college of Toulouse, relates that he was able, by this alternative alone, to save a woman who had become pregnant for the first time and at an advanced age. A few minutes after the section of the cervix uteri, the convulsions ceased, and the labour terminated spontaneously. Such a procedure, far from being blamable, ought, on the contrary, to be imitated under similar circumstances. Finally, when the dilatation of the uterine orifice has been effected spontaneously, or by the division of the cervix, if the labour be still too long protracted, we must lend a helping hand to nature. We must commence by rupturing the membranes, which alone is sometimes sufficient to allay the convulsions; for, by thus diminishing the volume of the uterus, its fibres are relaxed, its sensibility and irritability diminished; and the determination of blood to the head is obviated, by partly removing the impediment to its free circulation through the vessels of the abdomen. But, should the convulsions still continue, notwithstanding the evacuation of the amniotic fluid, we have no resource left but in the complete extraction of the fœtus." (P. 144.)

We are much surprised that M. Capuron does not recommend *venesection* in the rigid state of the os uteri above alluded to. He must surely be aware of the fact, that an os uteri which feels as hard as a board will often, after a copious bleeding, become as pliable as could possibly be wished. In some obstinate cases of this kind it is necessary to abstract a very large quantity of blood: this remedy, however, if used with sufficient vigour, is almost always effectual; and venesection, even to fifty ounces, is far preferable to so truculent a proceeding as slitting the cervix uteri, an operation which we can hardly conceive to be ever necessary, except in those deplorable, but fortunately rare cases, in which conception has taken place when the os uteri was affected with real scirrhus.

When speaking of compound pregnancy as a cause of manual interference, our author cites several ancient authorities with a gravity that is rather amusing; among others, that of Ambroise Paré, who relates that a woman brought forth twenty children at two deliveries. The credibility of this venerable surgeon on such matters may be estimated from the cases he has recorded of certain ladies, who, having been on rather too easy terms with the devil, or some of his satellites, became gravid by their good offices, and were, in due season, brought to bed of old clouts, hobnails, wood, stones, and a variety of heterogeneous and anormal products.* M. Capuron, however, judiciously concludes, that it is extremely rare for a woman to give birth even to three or four children at a time.

The portion of this work devoted to manual and instrumental labour is, in general, admirable. In considering the various irregular positions of the fœtus, the author has shown his good sense in discarding the frivolous minutiæ of classification in which continental writers have frequently indulged, and which only tend to burden the memory and bewilder the faculties of the student.

From this commendation we would except only the directions given for embryotomy. We wonder much that M. Capuron should inculcate the use of the *crotchet* in this operation, without so much as mentioning the forceps, which, in this country at least, are now very generally, and most advantageously substituted for it. The crotchet is an implement to which we can apply no other term than *detestable:* it is clumsy, of difficult application, often altogether insufficient for the desired end, and always fraught with infinite danger to the mother; while the embryotomy forceps, especially as improved by Drs. D. Davis and Conquest, are of more easy application, of more certain efficiency, and, if properly applied, perfectly safe to the parturient woman.

The reader may perhaps think that a work by so justly celebrated a practitioner as M. Capuron, on so important a branch of medical science, ought to have been the subject of a more extended notice than we have here given to it; but the fact is, that mere midwifery, disjoined from pathology, involves only subjects, on the greater part of which there can now scarcely be two opinions.

* Works, lib. xxv. c. 16. Johnson's Translation.

As a guide to the accoucheur in the mechanical department of his profession, this work will be found of great utility; but we will not deny that we think it much inferior, as a system of obstetrics, to those of several of our own countrymen, who have considered the mechanical details of the art in closer connexion with those pathological views from which they will be found inseparable in practice.

---

*The Edinburgh Medical and Surgical Journal, Nos.* CXVIII. *and* CXIX. *January and April,* 1834.

